# *N*-acetylglucosamine suppresses osteoclastogenesis in part through the promotion of *O*-GlcNAcylation

**DOI:** 10.1016/j.bonr.2016.02.001

**Published:** 2016-02-03

**Authors:** Tomoharu Takeuchi, Moyuko Nagasaka, Miyuki Shimizu, Mayumi Tamura, Yoichiro Arata

**Affiliations:** Faculty of Pharmaceutical Sciences, Josai University, Saitama 350-0295, Japan

**Keywords:** Gal, galactose, GalNAc, *N*-acetylgalactosamine, Glc, glucose, GlcNAc, *N*-acetylglucosamine, M-CSF, macrophage colony-stimulating factor, NF-κB, nuclear factor-κB, PBMC, peripheral blood mononuclear cell, PUGNAc, *O*-(2-acetamido-2-deoxy-_D_-glucopyranosylidene) amino *N*-phenylcarbamate, RANKL, receptor activator of nuclear factor-κB ligand, ROS, reactive oxygen species, sRANKL, soluble receptor activator of nuclear factor-κB ligand, TRAP, tartrate-resistant acid phosphatase, UDP, uridine diphosphate, Osteoclast, *N*-acetylglucosamine, GlcNAc, *O*-GlcNAcylation, NF-κB

## Abstract

Osteoclasts are the only cells in an organism capable of resorbing bone. These cells differentiate from monocyte/macrophage lineage cells upon stimulation by receptor activator of NF-κB ligand (RANKL). On the other hand, osteoclastogenesis is reportedly suppressed by glucose via the downregulation of NF-κB activity through suppression of reactive oxygen species generation. To examine whether other sugars might also affect osteoclast development, we compared the effects of monomeric sugars (glucose, galactose, *N*-acetylglucosamine (GlcNAc), and *N*-acetylgalactosamine (GalNAc)) on the osteoclastogenesis of murine RAW264 cells. Our results demonstrated that, in addition to glucose, both GlcNAc and GalNAc, which each have little effect on the generation of reactive oxygen species, suppress osteoclastogenesis. We hypothesized that GlcNAc might affect osteoclastogenesis through the upregulation of *O*-GlcNAcylation and showed that GlcNAc increases global *O*-GlcNAcylation, thereby suppressing the RANKL-dependent phosphorylation of NF-κB p65. Furthermore, an inhibitor of *N*-acetyl-β-_D_-glucosaminidase, *O*-(2-acetamido-2-deoxy-_D_-glucopyranosylidene) amino *N*-phenylcarbamate (PUGNAc), which also increases *O*-GlcNAcylation, suppressed the osteoclastogenesis of RAW264 cells and that of human peripheral blood mononuclear cells. Together, these data suggest that GlcNAc suppresses osteoclast differentiation in part through the promotion of O-GlcNAcylation.

## Highlights

• Along with glucose, the monomeric sugars GlcNAc and GalNAc suppress osteoclastic differentiation.
• Unlike glucose, GlcNAc and GalNAc have little effect on RANKL-induced ROS production.
• GlcNAc and the *N*-acetyl-β-_D_-glucosaminidase inhibitor PUGNAc both increase O-GlcNAcylation and suppress osteoclastogenesis.
• Upregulation of O-GlcNAcylation suppresses the RANKL-dependent phosphorylation of NF-κB p65.
• Together, these results suggest that GlcNAc suppresses osteoclastogenesis in part through the promotion of O-GlcNAcylation.

## 1 Introduction

Bone homeostasis is regulated by the balance between bone formation and resorption (Yamashita et al., 2012). Only a single bone-resorbing cell type is found in the body, known as osteoclasts (Cappariello et al., 2014). Osteoclasts are multinucleated cells that express tartrate-resistant acid phosphatase (TRAP) and differentiate from monocyte/macrophage lineage cells upon stimulation with macrophage colony-stimulating factor (M-CSF) and receptor activator of nuclear factor-κB (NF-κB) ligand (RANKL). M-CSF promotes the expression of RANK, the receptor of RANKL. RANKL stimulation activates downstream signaling molecules including NF-κB and c-Fos, which induce the expression of NFATc1, a master transcriptional regulator of osteoclast differentiation. In turn, NFATc1 induces the upregulation of osteoclast-specific genes including *TRAP*, cathepsin K, and matrix metallopeptidase 9 (Asagiri and Takayanagi, 2007, Kuroda and Matsuo, 2012, Boyce, 2013). Notably, these physiological differentiation processes are well reflected in the RANKL-dependent osteoclastogenic differentiation of RAW264 cells (Hsu et al., 1999).

Osteoclast differentiation is regulated by various molecules, including the monomeric sugar glucose (Glc) at high concentration. Glc suppresses osteoclastogenesis by suppressing the activity of NF-κB through an anti-oxidative mechanism, which entails suppression of the RANKL-induced generation of reactive oxygen species (ROS) (Wittrant et al., 2008), and suppressing the gene expression of several key differentiation molecules including NFATc1 (Xu et al., 2015). It has also been reported that a rare monomeric sugar, allose, inhibits osteoclast differentiation (Yamada et al., 2012). However, the effects of other common monomeric sugars such as galactose (Gal), *N*-acetylglucosamine (GlcNAc), and *N*-acetylgalactosamine (GalNAc) on osteoclastogenesis remain undetermined.

Glc is a well-known major energy source. Monomeric sugars including Glc are metabolized in cells and become activated as nucleotide sugars that are used for, e.g., *N*-glycosylation and *O*-GlcNAcylation (Freeze and Elbein, 2009). *O*-GlcNAcylation is the posttranslational modification of serine or threonine residues in various intracellular proteins by GlcNAc and is reversibly catalyzed by *O*-GlcNAc transferase and β-N-acetylglucosaminidase (*O*-GlcNAcase) (Butkinaree et al., 1800, Hanover et al., 1800). *O*-GlcNAcylation is thought to exhibit crosstalk with phosphorylation and has been shown to affect the activities of a variety of signaling molecules including those known to have important roles in osteoclastogenesis such as p38, ERK, NF-κB, c-Fos, and Akt (Butkinaree et al., 1800, Hanover et al., 1800).

In the present study, we compared the effects of simple sugars (Glc, Gal, GlcNAc, and GalNAc) on the RANKL-dependent osteoclastogenic differentiation of murine RAW264 cells. We also investigated the role of *O*-GlcNAcylation in this process by examining the effect of sugars thereon and the effects of an inhibitor of *N*-acetyl-β-_D_-glucosaminidase (PUGNAc), which increases *O*-GlcNAcylation, on RAW264 and human peripheral blood mononuclear cell (PBMC) osteoclast differentiation.

## 2 Materials and methods

### 2.1 Cell culture

The mouse macrophage-like RAW264 cell line was obtained from the RIKEN Cell Bank (Tsukuba, Japan) and maintained in modified Eagle's medium alpha (MEMα medium (Wako, Osaka, Japan) containing 10% heat-inactivated fetal bovine serum (Thermo Fisher Scientific, Waltham, MA, USA) and 1 × penicillin/streptomycin (Wako) under a humidified atmosphere containing 5% CO_2_ at 37 °C. Uncharacterized cryopreserved human PBMCs were obtained from Cellular Technology, Ltd. (Shaker Heights, OH, USA) and cultured in MEMα medium containing 10% heat-inactivated fetal bovine serum and 1 × penicillin/streptomycin.

### 2.2 Osteoclast differentiation and TRAP staining

RAW264 cells were seeded on a 96-well plate (1000 cells/well) and cultured for 1 day. Thereafter, the cells were treated with 250 or 500 ng/mL soluble RANKL (sRANKL) (Oriental Yeast, Tokyo, Japan) in the presence of 20 mM Glc, Gal, GlcNAc, GalNAc, or *O*-(2-acetamido-2-deoxy-_D_-glucopyranosylidene) amino *N*-phenylcarbamate (PUGNAc; an inhibitor of *N*-acetyl-β-_D_-glucosaminidase) (all from Wako) and allowed to differentiate for 4 days. The sugars were dissolved in phosphate-buffered saline (PBS); PUGNAc was dissolved in DMSO. Human PBMCs were seeded on a 96-well plate (1 × 10^5^ cells/well) and cultured for 1 day. Then the cells were treated with 50 ng/mL sRANKL and 25 ng/mL human M-CSF (PeproTech, Rocky Hill, NJ, USA) in the presence of 20 mM GlcNAc or 10 μM PUGNAc and allowed to differentiate for 8 days. Media were replenished every 2 days.

Differentiated cells were washed with PBS and then treated with 4% paraformaldehyde solution for 10 min at room temperature. After being washed again with PBS, the cells were treated with PBS and then stained with a TRAP staining solution containing 50 mM sodium tartrate, 45 mM sodium acetate, pH 5.2, 0.1 mg/mL naphthol AS-MX phosphate (Sigma-Aldrich, St. Louis, MO, USA), and 0.6 mg/mL fast red violet LB (Sigma-Aldrich), pH 5.2, for 1 h or longer at room temperature. The cells were viewed under a TC5400 microscope (Meiji Techno, Saitama, Japan) equipped with a Moticom 2000 digital camera (Shimadzu, Kyoto, Japan), and TRAP-positive cells that stained red and contained three or more nuclei were counted. Photographs were taken with a 10 × objective.

### 2.3 TRAP enzyme activity assay

RAW264 cells were allowed to differentiate as described in Section 2.2. After 4 days, the cells were washed with PBS and lysed with 100 μL TRAP buffer (50 mM sodium tartrate, 50 mM sodium acetate, 150 mM KCl, 0.1% TritonX-100, 1 mM sodium ascorbate, and 0.1 mM FeCl_3_, pH 5.2) for 10 min at 4 °C. The prepared cell extract (10 μL) was then added to 100 μL TRAP buffer containing 2.5 mM *p*-nitrophenyl phosphate (Thermo Fisher Scientific) as a TRAP substrate, and the reaction mixture was incubated for 1 h at 37 °C. After the addition of 50 μL 0.9 M NaOH to the mixture to stop the reaction, the absorbance at 405 nm was measured using a SpectraMax M5 microplate reader (Molecular Devices, Sunnyvale, CA, USA).

### 2.4 Real-time PCR

RAW264 cells were allowed to differentiate for 4 days as described in Section 2.2. Total RNA extraction and cDNA synthesis were performed using the Power SYBR® Green Cells-to-CT™ Kit (Life Technologies, Carlsbad, CA, USA) according to the manufacturer's instruction. In brief, cells differentiated in a 96-well plate were washed with PBS and lysed with 50 μL lysis solution containing DNase I. A portion of the lysate (10 μL) was used for reverse transcription with both random primers and oligo dT. Real-time PCR was performed with the StepOnePlus Real-Time PCR System (Applied Biosystems, Foster City, CA, USA) and the Power SYBR® Green PCR Master Mix (Life Technologies). All PCR products were amplified with 40 cycles of denaturation (95 °C, 15 s) and annealing and extension (65 °C, 15 s). Hypoxanthine guanine phosphoribosyl transferase (*Hprt*) was used as an internal control, and data were analyzed using the 2^−∆∆ Ct^ method. The primers used for PCR were as follows: *Hprt*, 5′-GCT CGA GAT GTC ATG AAG GAG-3′ and 5′-CAG CAG GTC AGC AAA GAA CTT-3′; cathepsin K, 5′-GGC TGT GGA GGC GGC TAT-3′ and 5′-AGA GTC AAT GCC TCC GTT CTG-3′; and matrix metallopeptidase 9, 5′-AAA GAC CTG AAA ACC TCC AAC CT-3′ and 5′-GCC CGG GTG TAA CCA TAG C-3′. The primers for *Hprt* were designed according to the method described by Nairn et al. (2008), and the others were according to that by Kim et al. (2014)).

### 2.5 Staining and measurement of ROS

For ROS staining, RAW264 cells were seeded on a 12-well plate (6000 cells/well) and cultured for 1 day. Then the cells were treated with 500 ng/mL sRANKL in the presence of 20 mM sugars and allowed to differentiate for 4 days. Differentiated cells were washed twice with PBS and then stained with PBS containing 5 μM fluorescent ROS detection reagent (5-(and-6)-chloromethyl-2′,7′-dichlorodihydrofluorescein diacetate, acetyl ester (CM-H_2_DCFDA); Life Technologies) for 30 min at room temperature. Thereafter, the cells were washed twice with PBS, and the resultant green fluorescence was viewed using a FLoid™ Cell Imaging Station (Life Technologies) with a 20 × objective.

For ROS measurement, RAW264 cells were seeded on a 96-well white plate (1000 cells/well) and cultured for 1 day. Then the cells were treated with 500 ng/mL sRANKL in the presence of 20 mM sugars and allowed to differentiate for 4 days. Differentiated cells were stained with PBS containing 10 μM CM-H_2_DCFDA for 1 h at room temperature. The cells were washed twice with PBS, and the fluorescent dye was extracted by incubation with 100 μL PBS containing 0.2% TritonX-100 for 30 min at 4 °C. Thereafter, the fluorescence (excitation 485 nm, emission 538 nm) was measured using a SpectraMax M5 microplate reader.

### 2.6 Western blotting

For immunoblotting analysis, cells were washed with PBS and lysed in 200 μL sample buffer (50 mM Tris–HCl, pH 6.8, 1% sodium dodecyl sulfate, 10% glycerol, 0.01% bromophenol blue, and 2% 2-mercaptoethanol) with sonication. After boiling and centrifugation, the resulting supernatants were subjected to sodium dodecyl sulfate-polyacrylamide gel electrophoresis, and the separated proteins were transferred onto nitrocellulose membranes (Bio-Rad Laboratories, Berkeley, CA, USA) using a wet electroblotting system for the detection of *O*-GlcNAcylated proteins or using the iBlot Gel Transfer Device (Life Technologies) for the other proteins of interest. Immunoblotting was performed on an iBind™ Western Device (Life Technologies) according to the manufacturer's instructions using horseradish peroxidase-conjugated anti-*O*-GlcNAc (CTD110.6) mouse monoclonal antibody, anti-IκBα mouse monoclonal antibody, anti-phospho-NF-κB p65 (Ser536) rabbit monoclonal antibody, and anti-NF-κB p65 rabbit monoclonal antibody (Cell Signaling Technology, Danvers, MA, USA). These antibodies were diluted ~ 1:1000 with 1 × iBind™ solution prior to use. The blots were visualized with a Luminata Crescendo (Merck Millipore), and signals were detected using a ChemiDoc XRS^+^ (Bio-Rad). Equal protein loading was confirmed by Coomassie brilliant blue staining.

### 2.7 Resorption assay

To measure resorption, RAW264 cells with less than three passages were seeded on an Osteoassay surface stripwell microplate (Corning, Cambridge, MA, USA) at a density of 2000 cells/well and cultured for 1 day. Then the cells were treated with 500 ng/mL sRANKL in the presence of 20 mM GlcNAc or 10 μM PUGNAc. Media were replenished every 3 days. After 14 days, the plate was incubated with 10% bleach to strip the cells, rinsed with distilled water, air dried, and imaged using a digital camera. The percentage of resorbed surface area was analyzed using NIH ImageJ software (Schneider et al., 2012).

Human PBMCs were seeded at a density of 1 × 10^5^ cells/well and cultured for 1 day. Then the cells were treated with 50 ng/mL sRANKL and 25 ng/mL M-CSF in the presence of 20 mM GlcNAc or 10 μM PUGNAc. Media were replenished every 2 days. After 14 days, the resorbed area was analyzed as described above.

### 2.8 Statistics

Data are presented as means ± S.D. Comparisons between two groups were analyzed using Student's two-tailed *t*-tests. p-values < 0.05 were considered statistically significant. Each experiment was repeated at least two times with similar results.

## 3 Results

### 3.1 Glc, GlcNAc, and GalNAc suppress the osteoclastic differentiation of RAW264 cells

**Fig. 1:**
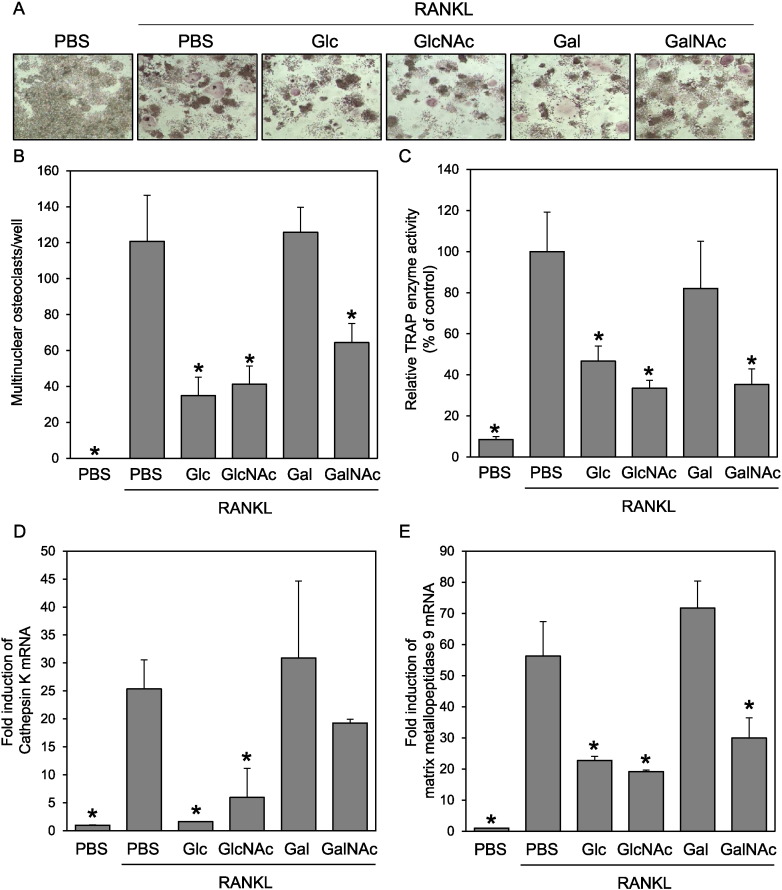
GlcNAc and GalNAc suppress the osteoclastic differentiation of RAW264 cells. RAW264 cells were treated with RANKL and various sugars. RANKL-treated cells were subjected to TRAP staining (A), and TRAP-positive multinuclear cells were counted (B) or were subjected to TRAP enzyme activity assay (C). (D and E) Real-time PCR analysis of the osteoclast marker genes cathepsin K (D) and matrix metallopeptidase 9 (E) using cDNA prepared from RANKL-treated RAW264 cells. Data are expressed as the means ± S.D. *p < 0.05 vs. control (PBS with RANKL).

To clarify the effect of simple sugars on the formation of osteoclasts, we stimulated RAW264 cells with RANKL in the presence of various sugars at 20 mM. We used equal high concentrations (20 mM) of sugars for osmotic control although the concentration of the sugars other than Glc is much higher than their physiological or pathological concentrations. After 4 days, TRAP-positive cells were stained (Fig. 1A), and TRAP-positive multinuclear cells were counted (Fig. 1B). Glc showed a suppressive effect on the formation of osteoclasts, as had been reported by Wittrant et al. (2008)), as did GlcNAc and GalNAc. In contrast, Gal showed little effect on osteoclast formation. To confirm these results, we performed a TRAP enzyme activity assay and found that Glc, GlcNAc, and GalNAc also suppressed RANKL-dependent upregulation of TRAP enzyme activity (Fig. 1C) without affecting cell viability (data not shown). We also examined the effect of 20 mM mannose on osteoclastogenesis; however, mannose showed cytotoxicity at this concentration (data not shown). Therefore, we excluded mannose from the subsequent experiments.

Next, to examine the effect of sugars on osteoclastogenesis at the molecular level, the expression of osteoclast marker genes was analyzed by real-time PCR. RAW264 cells were stimulated with RANKL in the presence of various sugars, and the mRNA expression of cathepsin K and matrix metallopeptidase 9 was analyzed after 4 days (Fig. 1D and E). RANKL stimulation induced the expression of these genes, whereas Glc, GlcNAc, and GalNAc suppressed their RANKL-dependent upregulation. Although the effects of GalNAc were relatively weak and its effect on cathepsin K gene expression was not statistically significant, GalNAc tended to suppress the expression of these genes. On the other hand, consistent with the results of TRAP-staining and the enzyme assay, Gal showed little effect on the expression of osteoclast marker genes.

### 3.2 GlcNAc and GalNAc suppress osteoclastic differentiation by different mechanisms than that used by Glc

As our results indicated that not only Glc but also GlcNAc and GalNAc could suppress the RANKL-dependent osteoclastogenesis of RAW264 cells, we next investigated the underlying molecular mechanisms. Wittrant et al. reported that high concentrations of Glc inhibited osteoclast formation, ROS production, and caspase-3 and NF-κB activity and suggested that the inhibition of redox-sensitive NF-κB activity through an anti-oxidative mechanism was important for the inhibition of osteoclast formation by Glc (Wittrant et al., 2008). Therefore, we compared the effects of sugars on ROS production.

**Fig. 2:**
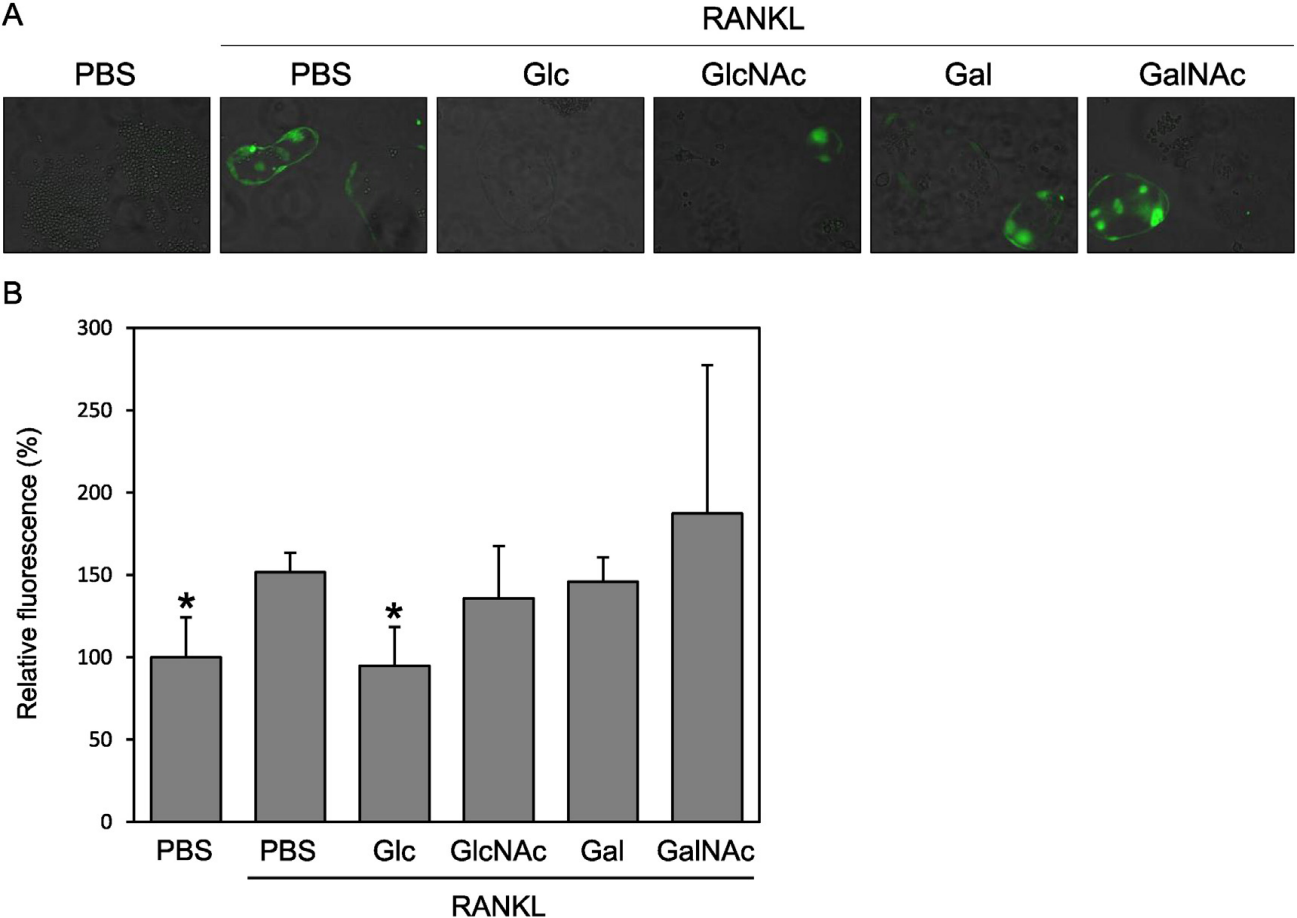
Among the tested monomeric sugars, only Glc suppressed the RANKL-dependent upregulation of ROS. RAW264 cells were treated with RANKL and various sugars. Intracellular ROS was detected using CM-H_2_DCFDA (A), and the resultant fluorescence intensity was measured (B). Data are expressed as the means ± S.D. *p < 0.05 vs. control (PBS with RANKL).

RAW264 cells were treated with RANKL in the presence of various sugars at 20 mM and then stained with the fluorescent ROS detection reagent CM-H_2_DCFDA followed by observation under a fluorescence microscope and quantification of fluorescent intensity (Fig. 2). From this, we determined that RANKL induced ROS generation whereas Glc suppressed its induction. In contrast, Gal, GlcNAc, and GalNAc showed little effect on RANKL-induced ROS generation. These results indicated that GlcNAc and GalNAc could suppress osteoclast formation through different mechanisms from that used by Glc, i.e., without inhibiting ROS generation.

### 3.3 Upregulation of *O*-GlcNAcylation suppresses osteoclastogenic differentiation partly through the inhibition of NF-κB p65 phosphorylation

Although both GlcNAc and GalNAc have suppressive effects on the osteoclastogenesis of RAW264 cells, the effect of GlcNAc is somewhat more substantial than that of GalNAc. Therefore, we focused on GlcNAc in the following experiments.

GlcNAc is metabolized intracellularly to UDP-GlcNAc, which is used for, e.g., *O*-GlcNAcylation and *N*-glycosylation (Freeze and Elbein, 2009). *O*-GlcNAcylation modifies numerous nucleocytoplasmic proteins and plays important roles in the regulation of various signaling pathways (Hart and Akimoto, 2009). The NF-κB signaling pathway, whose activation was shown to be important for osteoclastogenesis (Boyce, 2013), was found to be suppressed by the upregulation of *O*-GlcNAcylation (Xing et al., 2011, Zou et al., 2009). Therefore we investigated whether GlcNAc upregulates *O*-GlcNAcylation and whether such upregulation might affect osteoclastogenesis.

**Fig. 3:**
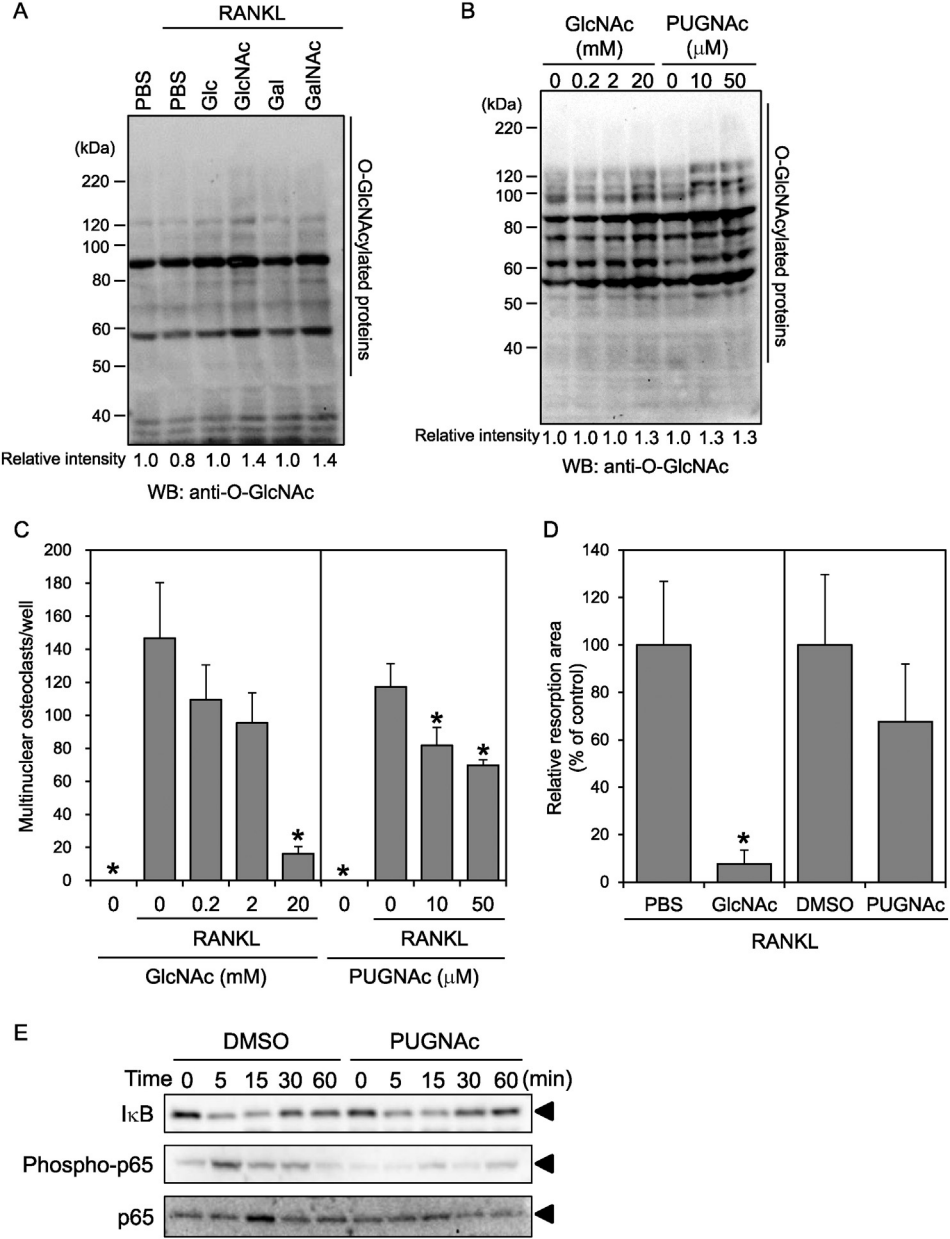
Upregulation of *O*-GlcNAcylation by GlcNAc and PUGNAc suppresses osteoclastic differentiation partly via suppression of NF-κB p65 phosphorylation. (A) RAW264 cells were treated with sRANKL in the presence of various sugars. Following 4 day differentiation, the cell lysates were subjected to western blotting with an anti-*O*-GlcNAc antibody. The intensities of total *O*-GlcNAcylated proteins were measured using ImageJ software. (B) RAW264 cells were treated with the indicated concentrations of GlcNAc or PUGNAc, an inhibitor of *O*-GlcNAcase, and subjected to western blotting with an anti-*O*-GlcNAc antibody. (C) RAW264 cells were treated with RANKL in the presence of the indicated concentrations of GlcNAc or PUGNAc. RANKL-treated cells were subjected to TRAP staining. (D) RAW264 cells were seeded on osteoclast assay surface plates and treated with RANKL in the presence of 20 mM GlcNAc or 10 μM PUGNAc. After 14 days, the resorption areas on the osteoclast assay surface plates were analyzed. (E) RAW264 cells were pretreated with PUGNAc or DMSO for 4 h and then stimulated with sRNAKL, harvested at the indicated time, and subjected to western blotting. Data are expressed as the means ± S.D. *p < 0.05 vs. control (PBS or DMSO with RANKL).

To clarify the effects of sugars on *O*-GlcNAcylation, RAW264 cells were treated with RANKL in the presence of various sugars at 20 mM and subjected to western blotting, and the *O*-GlcNAcylated proteins were then detected using an anti-*O*-GlcNAc antibody (Fig. 3A). We found that RANKL-stimulation in the presence of either GlcNAc or GalNAc resulted in a global increase of *O*-GlcNAcylation. This result suggested that the upregulation of *O*-GlcNAcylation suppressed osteoclast formation. Therefore, we next investigated the effect of increased *O*-GlcNAcylation on osteoclastogenesis. We found that the global *O*-GlcNAcylation of RAW264 cells increased when the cells were treated with GlcNAc or PUGNAc, an inhibitor of *N*-acetyl-β-_D_-glucosaminidase, which functions as an *O*-GlcNAcase (Fig. 3B). Furthermore, we investigated the effect of GlcNAc or PUGNAc on the formation of osteoclasts (Fig. 3C) and on the bone resorption activity of differentiated osteoclasts (Fig. 3D). We found that both compounds suppressed the number of formed osteoclasts and tended to suppress bone resorption activity, although the suppressive effect of PUGNAc was weaker than that of GlcNAc. These results indicate that the increase of *O*-GlcNAcylation suppressed the RANKL-dependent formation of osteoclasts from RAW264 cells.

The NF-κB signaling pathway is important for RANKL-dependent osteoclastogenesis. This pathway is reportedly suppressed by a global increase of *O*-GlcNAcylation through the inhibition of NF-κB p65 phosphorylation without an associated effect on IκB degradation (Xing et al., 2011). Therefore, we hypothesized that the suppression of NF-κB signaling by the increase of *O*-GlcNAcylation was one of the mechanisms underlying the suppressive effect of GlcNAc and PUGNAc on osteoclastogenesis. To test this hypothesis, RAW264 cells were pretreated with PUGNAc for 4 h and then stimulated with RANKL, followed by western blotting with anti-IκB, anti-phospho-p65, and anti-p65 antibodies (Fig. 3E). We found that RANKL stimulation induced the degradation of IκB and the phosphorylation of p65. However, pretreatment with PUGNAc suppressed the RANKL-dependent induction of p65 phosphorylation with little effect on IκB degradation. Pretreatment with GlcNAc showed similar results (data not shown), although the effect was less clear than that generated by PUGNAc, presumably because the increase in *O*-GlcNAcylation mediated by GlcNAc under this condition was smaller than that by PUGNAc (data not shown). Therefore, we concluded that *O*-GlcNAcylation upregulation could suppress the osteoclastogenic differentiation of RAW264 cells partly through the inhibition of NF-κB p65 phosphorylation, although other signaling pathways might also be affected by *O*-GlcNAcylation and have a role in the formation of osteoclasts.

### 3.4 Upregulation of *O*-GlcNAcylation suppresses the osteoclastogenesis of human PBMCs

**Fig. 4:**
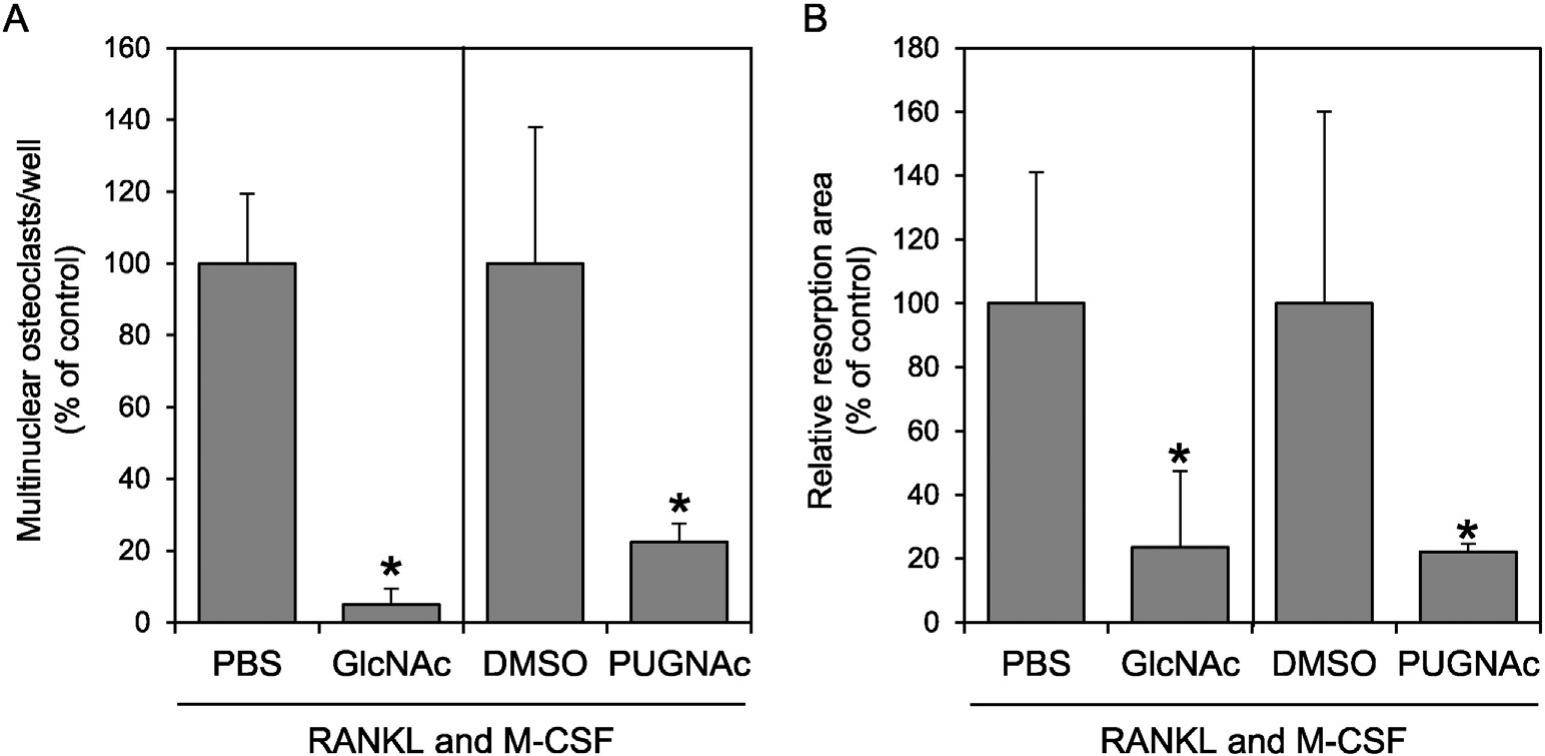
GlcNAc and PUGNAc suppress the osteoclastic differentiation of human PBMCs. (A) Human PBMCs were treated with sRANKL and M-CSF in the presence of GlcNAc or PUGNAc and allowed to differentiate for 8 days. TRAP-positive multinuclear cells were counted. (B) Human PBMCs seeded on osteoclast assay surface microplates were treated with sRANKL and M-CSF in the presence of GlcNAc or PUGNAc. After 14 days, the resorption areas on the osteoclast assay surface plates were analyzed. Data are expressed as the means ± S.D. *p < 0.05 vs. control (PBS or DMSO).

To determine the effect of increased *O*-GlcNAcylation under a more physiological condition, we treated human PBMCs with RANKL and M-CSF in the presence of GlcNAc or PUGNAc. The number of formed osteoclasts was examined by TRAP staining (Fig. 4A), and the activity of the osteoclasts was examined using a bone resorption assay (Fig. 4B). We found that both GlcNAc and PUGNAc suppressed osteoclast formation and activity. These results are consistent with the results obtained using RAW264 cells; therefore, we concluded that the upregulation of *O*-GlcNAcylation generally suppressed osteoclast differentiation. However, the effect of PUGNAc on PBMCs was more significant than that on RAW264 cells, and this difference might suggest that PUGNAc affects the early phase of differentiation, e.g., M-CSF-induced RANK expression.

## 4 Discussion

In the present study, using RAW264 cells as a model system, we compared the effects of simple sugars on osteoclastogenesis, although the concentration (20 mM) of the sugars other than Glc is much higher than their physiological or pathological concentrations. We found that GlcNAc and GalNAc suppressed osteoclastogenesis without affecting ROS generation, whereas the standard monomeric sugar Glc affected ROS generation as reported previously (Wittrant et al., 2008). These findings indicated different underlying molecular mechanisms for the suppression of osteoclastogenesis among these sugars. PUGNAc, an inhibitor of *O*-GlcNAcase, and GlcNAc both suppressed the osteoclastogenesis of RAW264 cells and human PBMCs. Supplemental GlcNAc is thought to couple with uridine diphosphate (UDP) to form UDP-GlcNAc, an activated form of GlcNAc that is utilized as a donor substrate for *O*-GlcNAcylation. Since supplemental GalNAc also somewhat increased *O*-GlcNAcylation in these cells, consistent with a report that UDP-GlcNAc is exchangeable with UDP-GalNAc (Freeze and Elbein, 2009), the suppressive effect of GalNAc on osteoclastogenesis might be due in part to the upregulation of *O*-GlcNAcylation. Furthermore, we showed that the upregulation of *O*-GlcNAcylation suppresses the RANKL-dependent phosphorylation of NF-κB p65. Since *O*-GlcNAcylation might modify the functions of various proteins (Butkinaree et al., 1800, Hanover et al., 1800), *O*-GlcNAcylation might therefore affect osteoclastogenesis not only via p65 phosphorylation but also through other mechanisms.

Recently, it was reported that a global increase of *O*-GlcNAcylation promoted the osteoblastic differentiation of MC3T3-E1 cells but had little effect on the osteoclast differentiation of RAW264 cells (Koyama and Kamemura, 2015). This seems inconsistent with our results; however, this discrepancy might be due to differences in the conditions used to induce differentiation such as cell density, RANKL concentration, and assay conditions. Furthermore, to assess differentiation, Koyama and Kamemura performed qRT-PCR of the *TRAP* gene and quantification of TRAP activity (Koyama and Kamemura, 2015), whereas we counted the number of formed osteoclasts and measured the bone resorption area. Since our assay assesses a later phase of differentiation, we might have been able to detect the suppressive effect of *O*-GlcNAcylation on osteoclast differentiation. In either case, it seems feasible that the upregulation of global *O*-GlcNAcylation that increases bone formation by promoting osteoblast differentiation would also decrease bone resorption by suppressing osteoclast differentiation. Therefore, the regulation of *O*-GlcNAcylation might serve as a target for treating bone diseases such as osteoporosis, and molecules such as GlcNAc or its derivatives that upregulate *O*-GlcNAcylation might become useful therapeutic agents.

The suppressive effect of GlcNAc on osteoclastogenesis was stronger than that of PUGNAc (Fig. 4C and D), although *O*-GlcNAcylation was equally increased by treatment with 20 mM GlcNAc or 10 μM PUGNAc (Fig. 4B). These results suggest that GlcNAc affects osteoclastogenesis not only through *O*-GlcNAcylation but also through other mechanisms. The activated UDP-GlcNAc resulting from cell metabolism of GlcNAc is used not only for *O*-GlcNAcylation but also for other types of glycosylation such as *N*-glycosylation. It has been reported that GlcNAc increases the branching of *N*-glycans and that this increase resulted in an increased affinity of *N*-glycan for galectins, a family of galactose-specific lectins (Lau et al., 2007, Johswich et al., 2014, Boscher et al., 2011). Since galectin-3 and -9 have suppressive activity on osteoclastogenesis (Li et al., 2009, Moriyama et al., 2014), GlcNAc might suppress osteoclastogenesis through an increase of *N*-glycan branching and the suppressive activity of galectins. In addition, given that *N*-glycan can be capped with sialic acid, and sialic acid and siglec-15, a sialic acid binding lectin, have been reported to have roles in osteoclastogenesis (Takahata et al., 2007, Hiruma et al., 2011, Ishida-Kitagawa et al., 2012, Kameda et al., 2013), GlcNAc might affect osteoclastogenesis by modulating the interaction between sialic acid and its binding partner(s). On the other hand, UDP-GlcNAc and UDP-GalNAc might be used for the biosynthesis of glycosaminoglycans. Since glycosaminoglycans also affect osteoclast differentiation (Ariyoshi et al., 2005, Shinmyouzu et al., 2007, Ariyoshi et al., 2008, Ling et al., 2010, Salbach et al., 2012), it is further possible that GlcNAc affects osteoclastogenesis through the modulation of glycosaminoglycan biosynthesis.

In conclusion, the results of the present study demonstrated that sugars in addition to Glc can affect osteoclast development and that GlcNAc suppresses osteoclastogenesis in part via the upregulation of *O*-GlcNAcylation, although other mechanisms might also be involved, as described above. Further studies into these processes might reveal a new mechanism for the regulation of osteoclast differentiation by sugar molecules. In turn, this might provide new strategies for the development of clinical treatments or preventative measures for bone diseases.

## Conflict of interest

None.

## Figures and Tables

**Supplemental Fig. 1:**
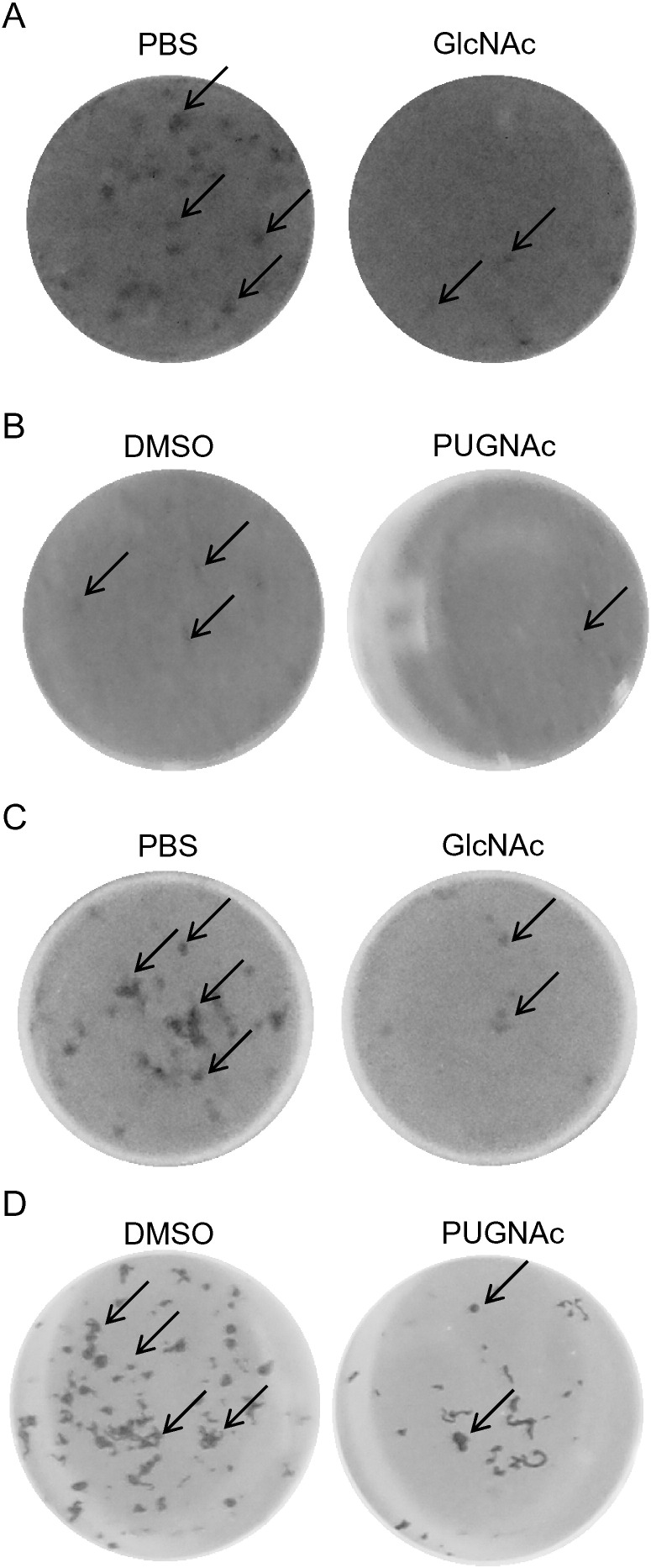
Resorption pits generated by RAW264 cells and PBMCs. (A and B) Representative images of resorption pits generated by RAW264 cells treated with RANKL and either GlcNAc (A) or PUGNAc (B). These images correspond to the data presented in Fig. 3D. (C and D) Representative photos of resorption pits generated by PBMCs treated with RANKL, M-CSF, and either GlcNAc (C) or PUGNAc (D). These images correspond to the data presented in Fig. 4B. Some, but not all, generated pits are indicated by arrows.
